# Notice of redundant publication: Aerobic capacity and disease activity in children, adolescents and young adults with juvenile idiopathic arthritis (JIA)

**DOI:** 10.1186/1546-0096-11-2

**Published:** 2013-01-14

**Authors:** Alberto Martini, Charles Spencer

**Affiliations:** 1 Editors-in-Chief, Pediatric Rheumatology

## 

## Comment on

**Philomien A Pelt**, **Tim Takken**, **Marco Brussel**, **Inge Witte**, **Aike Kruize** and **Nico M Wulffraat**

Aerobic capacity and disease activity in children, adolescents and young adults with juvenile idiopathic arthritis (JIA).

*Pediatric Rheumatology 2012, ****10****:27 doi:*10.1186/1546-0096-10-27

URL http://www.ped-rheum.com/content/10/1/27/

The Editors-in-Chief would like to alert readers that due to an error during the publication process this article [1] was published twice. The original article can be found in this journal [2].
